# Improving screening and management of latent tuberculosis infection: development and evaluation of latent tuberculosis infection primary care model

**DOI:** 10.1186/s12879-021-06925-8

**Published:** 2022-01-12

**Authors:** Marina Kunin, Mark Timlin, Chris Lemoh, David A. Sheffield, Alana Russo, Shegofa Hazara, Jacqueline McBride

**Affiliations:** 1 Monash Health Refugee Health and Wellbeing, Monash Health, 122 Thomas Street, Dandenong, VIC 3175 Australia; 2 Monash Infectious Diseases, Melbourne, VIC Australia

**Keywords:** Latent tuberculosis infection, Primary care, Refugee health, Culturally and linguistically diverse populations, Health care evaluation, Health care delivery, Health plan implementation, Patient education

## Abstract

**Background:**

In Australia, demand for specialist infectious diseases services exceeds capacity to provide timely management of latent tuberculosis infection (LTBI) in areas of high refugee and asylum seeker settlement. A model for treating LTBI patients in primary care has been developed and piloted in a refugee-focused primary health service (Monash Health Refugee Health and Wellbeing [MHRHW]) and a universal primary care clinic. This study reports on the development and evaluation of the model, focusing on the model feasibility, and barriers and enablers to its success.

**Methods:**

A convergent mix-methods design was used to evaluate the model for treating LTBI patients in primary care, where a prospective cohort study of patients commencing treatment either at MHRHW or the universal primary care clinic determined the model feasibility, while focus groups with clinicians directly involved in treating these patients explored barriers and enablers to sustainability and success of the model.

**Results:**

From January 2017 to April 2018, 65 patients with confirmed LTBI presented at participating clinics. Treatment was accepted by 31 (48%) patients, of whom 15(48%) were treated at MHRHW and 16 (52%) at the universal primary care clinic. The 6-months’ treatment completion rate was higher at MHRHW compared to the universal primary care clinic (14 (93%) compared to 9 (56%) respectively, p = 0.0373). Reasons for non-completion included adverse reaction, opting out and relocation. At the completion of the pilot, 15 clinicians participated in two focus groups. Clinicians identified barriers and enablers for successful LTBI management at patient, provider, organisational and clinical levels. While barriers for treatment completion and adherence were consistent across the two pilot sites, enablers, such as resources to facilitate patient education and follow-up, were available only at MHRHW.

**Conclusion:**

Screening and management of LTBI patients can be achieved within the primary care setting, considerate of barriers and enablers at patient, provider, organisational and clinical levels. Upscaling of a primary care response to the management of LTBI will require supporting primary care clinics with resources to employ dedicated clinical staff for patient education, follow-up communication and monitoring medication adherence.

**Supplementary Information:**

The online version contains supplementary material available at 10.1186/s12879-021-06925-8.

## Background

Approximately one quarter of the world’s population is estimated to have latent tuberculosis infection (LTBI), an asymptomatic infection with *Mycobacterium tuberculosis* [1]*.* For people with LTBI, the lifetime risk of developing the active disease is approximately 5–10% [2]. Preventive treatment of LTBI is recommended to reduce the risk of progression to active tuberculosis (TB) and further transmission of mycobacteria in settings with low TB prevalence, where re-exposure risk is low [3, 4].

In Australia, TB incidence rate is approximately five cases per 100 thousand population, and the majority of TB infection occurs due to LTBI reactivation in people who were born overseas [5, 6]. Refugees and asylum seekers are at particular risk of LTBI, as they frequently originate from countries with high TB incidence, and experience further exposure to *Mycobacterium tuberculosis* when travelling to host countries [7]. Diagnosing and treating refugees and asylum seekers with LTBI in the early stages of their settlement is important to both improve the health and wellbeing of these community groups, while also containing the risk of TB re-emerging within the broader Australian population. Therefore, it is recommended that refugees and asylum seekers are screened for LTBI within 1 month of their arrival in Australia [8]. Similar to other countries that resettle refugees through the United Nations High Commissioner for Refugees (UNHCR) program, screening and treatment of LTBI in refugees and asylum seekers commonly occurs in refugee health clinics, specialist infectious diseases clinics and hospital settings [9–13]. Traditional models of care, however, are associated with low treatment completion rates [14], Recognising the weaknesses of traditional models of care, the End TB Strategy calls to invest in research and new tools to allow equitable access to diagnosis and care [15].

Since 2009, the number of refugees and asylum seekers reaching Australia annually has varied between 13,000 to 20,000 [16]. Approximately 30% to 40% of these humanitarian arrivals are resettled in Victoria, with the City of Greater Dandenong in the south-eastern region of Melbourne being home to the largest community of asylum seekers and refugees within the state [17]. In areas of high settlement, such as Greater Dandenong, demand for specialist tertiary services frequently exceeds service capacity. Consequently, on-arrival screening does not always result in timely follow-up after LTBI detection, which is crucial to implementing required treatment [9, 12, 18].

Previous studies have suggested that shifting screening and management of LTBI from specialist settings towards primary care offers an effective strategy for potentially identifying people at high risk of developing TB [19] and may reduce diagnostic delay and improve the delivery of treatment [20]. Furthermore, General Practitioner (GPs) have expressed a high level of support for a primary care based model of care, provided there is appropriate training and support systems in place for participating staff [21]. However, literature describing and evaluating LTBI primary care models is limited. This paper aims to address this gap in the evidence base by describing the Latent Tuberculosis Infection Primary Care Model and presenting the evaluation findings associated with this initiative.

## Methods

### Intervention: latent tuberculosis Infection (LTBI) primary care model

#### Context and setting

Two health services were directly involved in the development and implementation of the LTBI Primary Care Model:

1. A local, universal primary care clinic was engaged to pilot this initiative. Located in central Dandenong, this clinic provides primary care services to the diverse Dandenong population, including a high proportion of patients from refugee backgrounds. The universal primary care clinic has an onsite pharmacy and is a private practice where consultations are subsidised through Australia’s universal healthcare insurance scheme, Medicare, with no out-of-pocket consultation fee incurred for most patients. In addition, this clinic is largely staffed by international medical graduates, many of whom are bilingual and able to speak the languages of the culturally and linguistically diverse community within the region.
2. Monash Health Refugee Health and Wellbeing (MHRHW) is a State funded refugee-focused health service, located in a community health facility in central Dandenong. MHRHW provides integrated primary care and specialist services, including an infectious diseases clinic. The service does not replicate universal primary care clinics, but rather provides intensive transitional care to asylum seekers and refugees experiencing high levels of vulnerability, complex health needs and restricted access to Medicare. Defining features of the MHRHW service model include: initiation and completion of refugee health assessments; extensive use of interpreting services; and, delivery of capacity building and community development strategies. MHRHW is comprised of a multi-disciplinary team, including GPs, GP refugee health fellow, refugee health nurses (RHNs), infectious diseases physicians, paediatricians, bicultural workers, community development workers, psychiatrists, counsellors and pharmacists. The GP refugee health fellow and one of the RHNs are experienced in the management of LTBI and were fundamental to the development and implementation of the LTBI Primary Care Model.

MHRHW patients are routinely screened for LTBI and, historically, treatment has been managed internally by the specialist infectious diseases outpatient clinic, with medication provided by the Victorian TB Program through hospital pharmacies at no cost to the patient. However, an increase in refugee and asylum seeker settlement in the south-eastern region of Melbourne over successive years resulted in additional demand for infectious diseases outpatient services, outstretching the capacity to deliver timely management of LTBI [17].

In consultation with the Victorian TB Program and the Continuous Professional Development (CPD) program of Royal Australian College of General Practitioners (RACGP), MHRHW designed a model of care to support the management of LTBI within the primary care setting. This model was intended to have broad applicability, relevant and feasible for both refugee-focused health services and universal primary care services.

**Fig. 1:**
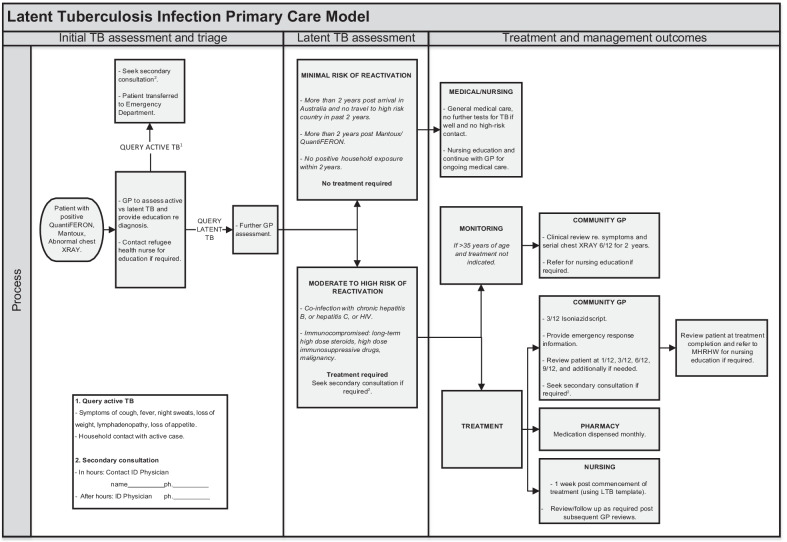
Latent Tuberculosis Infection Primary Care Model: a process of initial TB assessment and triage, latent TB assessment, and treatment outcomes

Figure 1 depicts the LTBI Primary Care Model flowchart.

In accordance with the model, individuals who screened positive for QuantiFERON, Mantoux, and/or had abnormal chest XRAY changes consistent with inactive TB, were assessed and treated by primary care providers at either MHRHW or the universal primary care clinic. Support and secondary consultations were available from MHRHW infectious diseases physicians. At the universal primary care clinic, access to the secondary consultations was facilitated by biweekly conference calls with the trained RHN and GP refugee health fellow. While the Victorian TB Program provided isoniazid free of charge to all patients, co-prescription of vitamin B6 and associated dispensing costs for patients attending the universal primary care clinic were offset by a grant from the South Eastern Melbourne Primary Health Network (SEMPHN). MHRHW patients had free access to the medication through the onsite pharmacy.

In accordance with LTBI management guidelines [22], patients were offered 9-months’ treatment with isoniazid monotherapy (300 mg daily), although 6-months’ was accepted as treatment completion. At the time of the study, isoniazid was the only treatment for LTBI listed on Pharmaceutical Benefits Scheme (PBS) in Australia. This medication may cause hepatitis, gastrointestinal, dermatological and neuropsychiatric adverse reactions, but is generally well tolerated [23]. However, the risk of isoniazid-related hepatotoxicity rises with age and therefore, treatment with isoniazid is applied with caution in patients over 35 years old. To reduce the risk of peripheral neuropathy [6], a co-prescription of vitamin B6 (pyridoxine, 25 mg daily) was added to the isoniazid.

To support effective management of patients through the LTBI Primary Care Model, MHRHW developed a comprehensive training module for primary care providers, GPs, and nurses, consistent with the RACGP’s CPD requirements. The training included both theoretical and practical modules, with a focus on:

- Recognising patients with LTBI that can be successfully managed in the community without specialist referral;
- Understanding clinical implications of LTBI management options: TB preventative treatment versus observation only;
- Providing culturally appropriate education to patients to differentiate between latent and active TB;
- Understanding and implementing a model of care incorporating nursing staff in the management of LTBI;
- Understanding potential complications of LTBI therapy.

To support effective patient education and follow-up, a Patient Education Resource Pack (Additional file 1) was developed, comprising of:

- Patient Booklet, which included pictures, a timetable and emergency contact information;
- Nurse Patient Education Checklist; and,
- Nurse Patient Follow-up Template.

### Study of the intervention

#### Design

A convergent mixed-methods design was applied, in which qualitative and quantitative data were collected and organised separately to provide complementary evidence to answer the research question [24]. A prospective cohort study of patients commencing treatment either at MHRHW or the universal primary care clinic was conducted to determine the feasibility of treating LTBI patients at each of these primary care sites; focus groups with clinicians directly involved in treating patients with LTBI at either MHRHW or the universal primary care clinic were conducted to explore the barriers and enablers to sustainability and success of the LTBI Primary Care Model at each of the participating sites. Findings were organised following the SQUIRE 2.0 guidelines [25].

#### Data collection and measures

##### Participants

###### Patients

Asylum seekers and refugees between the ages of 18–50 years, who screened positive for QuantiFERON, Mantoux, and had normal chest XRAY or abnormal XRAY changes consistent with inactive TB (lung granuloma or stable lung scarring), were eligible to participate in this study. Patients were excluded if they reported a history of TB treatment in their initial screening, had chest XRAY changes suspicious for active pulmonary TB, were pregnant, had complex comorbidities, or were considered unable to provide informed consent. Patients who met the inclusion criteria and accepted treatment were assigned by direct selection to be treated at either MHRHW or the universal primary care clinic.

###### Clinicians

1. GPs and nurses from MHRHW and the universal primary care clinic who were directly involved in providing care to the enrolled patients.
2. Infectious diseases physicians, GP refugee health fellow and RHN at MHRHW who trained and supported GPs and nurses involved in the LTBI Primary Care Model.

##### Data collection

The following data were collected:

1. Patient demographics, reasons for declining or not completing the treatment, compliance, adverse reactions and requirement for a secondary consultation with infectious diseases physicians were collected from January 2017 to April 2018. Data were collected by the treating clinician during monthly follow-up and recorded in an Excel spreadsheet.
2. Data on medication dispensing were recorded by the pharmacy. For the purpose of this study, a dispensing record of at least 6-months’ was considered as treatment completion [6, 26, 27].
3. Qualitative data collection occurred between August–September 2019. This involved conducting two 1-h focus group discussions; one with MHRHW and another with the universal primary care clinic clinicians. Clinicians unable to physically attend the focus group were invited to phone-in. An experienced qualitative researcher (MK) facilitated the discussions, while a second non-participant observer, a GP and researcher who works at MHRHW (MT), noted details of non-verbal communication, contextual issues, and the strength of emotional responses. The focus groups were guided by a theme list with prompting questions to explore clinicians’ experiences in implementing the LBTI Primary Care Model, including: training; utilisation of infectious diseases physician support; challenges of patient engagement; barriers and enablers to treatment completion; and perceived sustainability and expansion potential. Clinicians were also encouraged to discuss other issues they saw as relevant to the evaluation. All focus group discussions were audio recorded and then summarised according to the topics discussed.

##### Analysis

Descriptive statistics were used to calculate patient demographic and clinical characteristics including the proportion of patients commencing and declining medical treatment for LTBI, adherence to prescribed treatment, premature cessation of treatment, adverse reactions to treatment, consultation with infectious diseases physicians and completion rate of planned treatment. A two-tailed Fisher's test using GraphPad software was performed to examine the difference in treatment completion rates at MHRHW and the universal primary care clinic.

Qualitative exploratory descriptive analysis [28] oriented toward summarising the informational contents of the data to understand the perspectives of the service providers involved in the implementation, was used to analyse the focus group data. The analytical process was iterative. First, the written summaries of the audio recordings were reviewed, and data were categorised into themes defined as relevant for the evaluation of clinicians’ experiences in the pilot. A list of data-driven codes was then generated, and the pre-existing categories were modified to accommodate new insights from the data. Data saturation was assumed when no new codes and/or themes were emerging from the data [29]. The codes and categories were then reviewed by all members of the research team. Slight modifications were made to the coding structure to better align with the research aims. Interpretation disagreements were resolved by consensus. Data organisation was assisted by NVivo12 software.

The study was approved by Monash Health Human Research Ethics Committee HREC/17/MonH37.

## Results

### Patient data

From January 2017 to April 2018, 65 LTBI positive patients presented at participating services. Treatment was accepted by 31 (48%) patients. Common reasons for treatment not being offered or being declined by patients included: age older than 35; medical contraindications including pregnancy or breastfeeding; and, personal or social difficulties. Table 1 presents the demographic characteristics of participants and those who declined treatment.

**Table 1:** Patient demographic characteristics

	Accepted treatment n = 31 (48%)	Declined treatment n = 34 (52%)
Gender:		
Female	19 (61%)	14 (41%)
Male	12 (39%)	20 (59%)
Mean age	30	34
Country of origin		
Afghanistan	17 (55%)	17 (50%)
Iran	3 (10%)	1 (3%)
Ethiopia	3 (10%)	1 (3%)
Malaysia	1 (3%)	3 (9%)
Sri Lanka	1 (3%)	2 (6%)
Burma	1 (3%)	2 (6%)
Indonesia	1 (3%)	1 (3%)
Tibet	1 (3%)	N/A
Sudan	1 (3%)	N/A
Pakistan	1 (3%)	N/A
Syria	1 (3%)	N/A
South Sudan	N/A	4 (12%)
Iraq	N/A	1 (3%)
Nigeria	N/A	1 (3%)
Kenya	N/A	1 (3%)

Of the 31 patients who commenced treatment, 23 (74%) completed treatment by collecting at least 6-months of medication according to pharmacy dispensing records. Adverse reactions to treatment were experienced by 12 (39%) patients. Secondary consultation with an infectious disease physician was required for 11 (35%) patients, and with this support, all of these patients’ care continued.

#### MHRHW and universal primary care clinic comparison

Of the 31 LTBI patients who accepted treatment, 15 (48%) were treated at MHRHW and 16 (52%) at the universal primary care clinic. The patients at MHRHW and the universal primary care clinic were of similar age (mean age 31 and 30 respectively), and sex distribution (8 (53%) and 8 (50%) female respectively). Comorbid conditions recorded in patients treated at MHRHW included Vitamin D deficiency in two patients, and Hepatitis B plus Vitamin D deficiency in two other patients. For patients who were treated at the universal primary care clinic, two had Vitamin D deficiency.

**Fig. 2:**
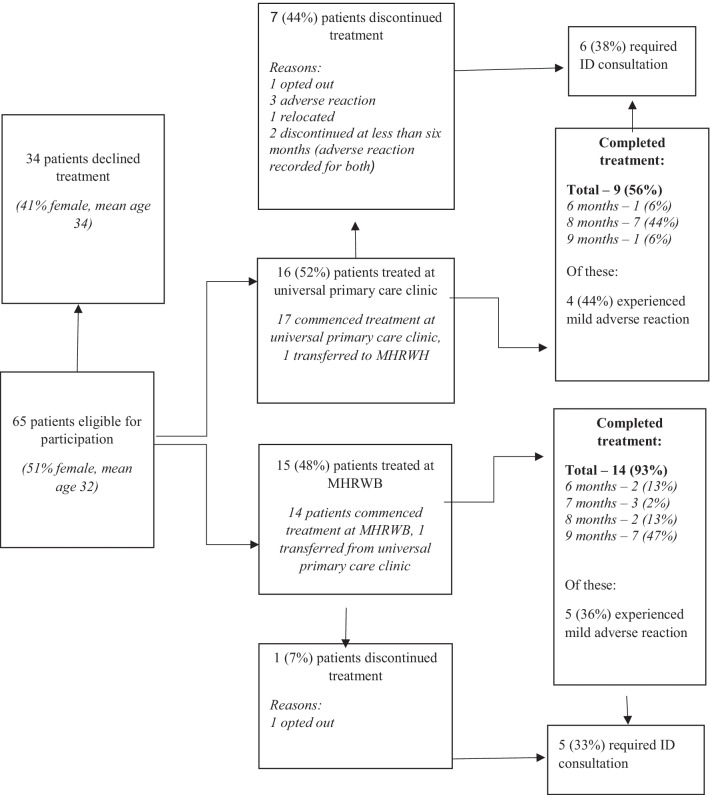
Patient pathway flowchart

The 6-months’ treatment completion rate was higher at MHRHW compared to the universal primary care clinic (14 of 15 (93%) patients compared to 9 of 16 (56%) patients respectively, p = 0.0373). One patient relocated from the universal primary care clinic to MHRHW and is included in the MHRHW data. The reasons for the universal primary care clinic patients not completing treatment included adverse reactions to the medication (three patients), discontinued collecting their medication (two patients), a personal decision to opt-out (one patient) and relocation (one patient). One patient from MHRHW did not complete treatment due to a personal decision to opt-out. Mild adverse reactions that did not preclude treatment completion were recorded for four (44%) patients at MHRHW and five (36%) patients at the universal primary care clinic. Secondary consultation with an infectious diseases physician was required by similar proportions of patients at both sites; five (33%) at MHRHW and six (38%) at the universal primary care clinic. Figure 2 outlines the patient treatment pathway.

### Provider perspectives on barriers and enablers for LTBI primary care model sustainability

At the completion of the pilot period, 15 clinicians that were directly involved in implementing the model of care participated in two focus groups. Six clinicians from the universal primary care clinic (abbreviated as U) participated in the first focus group, and nine clinicians from MHRHW (abbreviated as M) participated in the second focus group. Two of the participants were nurses, 8 GPs, 3 infectious diseases physicians and 2 pharmacists.

Clinicians described perceived barriers and enablers to the long-term adoption of the piloted model of care, and these were subsequently categorised by researchers into ‘patient’, ‘provider’, ‘organisational’ and ‘clinical’ level influences (see Table 2).

**Table 2:** Perceived barriers and enablers to the long-term adoption of the LTBI primary care model

	Barriers	Enablers
Patient-level	Low motivation to engage with the treatment Difficulty in processing diagnosis-related information Competing priorities	Patient education sessions delivered by trained RHN both prior to, and as required throughout, treatment
Provider-level	Low confidence in identifying and responding to adverse reactions caused by LTBI medication	Participation in high quality primary care provider education sessions Ongoing support from MHRHW infectious diseases physicians Co-design and established relationships with infectious diseases physicians Familiarity with cultural and clinical aspects relevant to the refugee patient group Multilingual proficiency and interpreter access
Organisational-level	Operational limitations including: Limited time for patient education and follow-up Lack of workforce to coordinate patient education and follow-up Limited financial resources	Extensive involvement of MHRHW RHN in patient follow-up Extended GP consultations Designated resources and time to support patient education and clinical follow-up Resources to proactively contact patients who fail to attend appointments or pick up medication
Clinical-level	Available treatment barriers: Adverse reactions Length of treatment	Free access to medication

#### Patient-level barriers and enablers

In relation to patient-level barriers, clinicians at both participating sites described experiencing challenges with patient engagement and follow-up to monitor side effects and ensure medication compliance: “From the GP perspective, you could manage the patients, but the follow-up was difficult, they do not turn up” (04U, GP).

Clinicians indicated that patients’ motivation to engage with LBTI treatment was limited, and this contributed towards difficulties in long-term patient engagement and follow-up. Some clinicians believed that the asymptomatic nature of the condition and the extended duration of treatment made it difficult for patients to prioritise and comply with their treatment schedule. In addition, clinicians observed that many patients struggled to process diagnosis-related information. A bicultural RHN explained: “In our [Afghan] culture there is no such thing as latent TB, you just have TB” (04 M, nurse). One participating GP described how difficulties in processing health information can become a cause of stress for some refugee and asylum seeker patients: “Even if patients say that they understand, this may not necessarily be the case and they go home quite traumatised” (06 M, GP). Competing priorities were also described as a barrier to patient engagement as refugee patients “have many other things to worry about” (04 M, nurse).

However, clinicians expressed that these identified patient-level barriers could, in part, be mitigated through routine patient education. Patient education was a core component of the LTBI Primary Care Model, with structured sessions delivered by a trained clinician and supported by the Patient Education Resource Pack (Additional file 1). This was readily achieved at MHRHW, where a trained RHN had allocated time to LTBI education and follow-up. These resources could not be replicated at the universal primary care clinic, and even though the MHRHW RHN nurse also delivered education sessions at the universal primary care clinic, patient education and follow-up remained challenging. In addition, a number of factors were identified as contributing to the successful education of patients, these included: use of the “teach-back” technique—a communication method used to confirm that a patient understands what has been explained to them and can relay the information back to the health provider accordingly: “I was asking them “tell me, what do you think you have?” (04 M, nurse); distributing the information about LTBI across a number of education sessions: “building knowledge in separate sessions worked well” (04 M, nurse); and utilising visual aides to support spoken messages: “It was helpful as patients had something in front of them and a number they can call. I had a lot of calls” (04 M, nurse).

#### Provider-level barriers and enablers

*Low confidence* of GPs in responding to medication-related adverse reactions was described as a barrier in LTBI patient management. GPs felt that they were “more cautious” (04U, GP) about the adverse reactions than infectious diseases physicians would be, and “when patients had side effects from the medication … decided to stop the medication and contact [infectious diseases] staff who would advise us what to do” (02U, GP). Adverse reactions from the medication were described as the most frequent reason for seeking infectious diseases physicians’ opinion at both participating sites.

Barriers at the provider-level were, in part, mitigated by comprehensive LTBI training and ongoing support from MHRHW infectious diseases physicians. Primary care providers at both participating sites described the training as “intensive”, “of high standards” and “holistic”. However, they also acknowledged that “everything cannot be covered during training” (04U, GP), and therefore appreciated having infectious diseases physicians available for consultation as required and biweekly conference calls with the GP refugee health fellow and RHN. From the perspective of infectious diseases physicians, primary care clinicians’ training and support was a “two-side experience” (09 M, infectious diseases physician) and “an opportunity to learn about ways LTBI might be more effectively and safely managed in a community-based setting” (09 M, infectious diseases physician).

Another facilitative factor mentioned by the participants was that the initiative evolved through a process of codesign and established relationships. Clinicians reflected positively on having “…built this project together” (01 M, infectious diseases physician), as this was considered pivotal in promoting effective communication amongst treating clinicians. Furthermore, clinicians at both participating sites identified cultural competency and familiarity with refugee health as an additional facilitating factor. One GP explained that clinical and cultural aspects of treating LTBI were “not new to us because we are familiar with the refugee group” (04U, GP). It was also highlighted that many primary care providers at both participating sites were able to communicate with patients in their first language or had access to interpreting services, which further nurtured effective management.

#### Organisational-level barriers and enablers

Resource availability, such as extended consultations and staffing resources, was described as an important factor, influencing both the delivery of clinical care and treatment outcomes. In particular, a dedicated resource for patient education and follow-up was considered paramount. MHRHW clinicians believed that extensive involvement of a RHN in driving patient follow-up nurtured patient engagement and facilitated treatment completion. The RHN, in consultation with infectious diseases physicians, developed the Patient Education Resource Pack (Additional file 1), which was believed to substantially contribute to successful patient engagement and follow-up. Application of these resources was described as limited at the universal primary care clinic as there were no dedicated staff to coordinate patient follow-up.

Financial viability of managing LTBI patients in universal primary care was also discussed. Despite GPs billing LTBI consultations as chronic illness consultations, utilising GP management plans and Team Care Arrangements [30], it was believed that the Medicare billing model was not adequate in renumerating for the time spent “to call, to follow-up and chase [patients] up” (05 M, GP). It was acknowledged that some challenges in patient follow-up and medication adherence would have been resolved through longer consultations. One infectious diseases physician explained: “[when] patients have more time to talk, you will find out if the patient is not taking medication” (10 M, infectious diseases physician).

#### Clinical-level barriers and enablers

Limitations of the LTBI medication (isoniazid), such as adverse reactions and the length of treatment, were described as clinical-level barriers. Adverse reactions to the medication were believed to be the main barrier to treatment completion. A nurse explained: “[the patients] take the first batch of the medication, they complain about side effects and they stop” (07 U, nurse). The length of treatment was described as another barrier, with some clinicians believing that the lengthy treatment hindered patient compliance.

Treating clinicians from the universal primary care clinic emphasised the importance of free medication in facilitating treatment completion: “to pay [for the medication] would be a problem [for our patients]” (03 U, pharmacist).

## Discussion

Our results on treatment completion and adherence at both pilot sites demonstrate that LTBI can be reasonably and effectively managed within primary care setting. Barriers for treatment completion and adherence, which potentially can influence the sustainability of the proposed model, were consistent across the two pilot sites; however, enablers varied with key determinants of success proposed.

The results of the study suggest that some barriers for treating LTBI patients, such as patients’ low levels of motivation, difficulty in processing diagnosis-related information and competing priorities, along with the limitations of LTBI medication (isoniazid), such as the length of treatment and adverse reactions, appear to be relevant regardless of the treatment setting. For example, challenges with patient compliance have been reported in prior studies where treatment was conducted within the tertiary sector, with treatment completion rate being around 45–60% [31, 32]. Evidence from previous studies also confirms that medication intolerance is a significant variable impacting treatment completion [33] and that treatment completion rate can be improved by introducing shorter regiments [34, 35]. While isoniazid is currently the only treatment for LTBI listed on PBS in Australia [6], an alternative 4-month therapy with rifampicin is becoming an accepted option as it has shown higher rates of treatment completion and better safety [27, 36]. Additionally, a 3-month regimen of once-weekly isoniazid combined with rifapentine is accepted, however, rifapentine currently has extremely limited availability in Australia [27]. Introducing rifamycin based treatment with shorter regiments as the first line LTBI treatment should be recommended in Australia as this can improve LTBI treatment completion rates.

In the current study, low level of primary care clinicians’ confidence in recognising and responding to adverse reactions to medication, particularly in patients with additional medical and social issues, was mitigated through the ongoing support from infectious diseases services in the form of training and secondary consultations. These findings suggest that the confidence of primary care clinicians could be improved by extended support from infectious diseases physicians and more in-depth reference resources, with multiple anticipated problems and suggested solutions, for the primary care clinicians to refer to during the patient consultation. Presumably, however, as primary care providers acquire more experience in managing LTBI patients, their dependence on infectious diseases physicians’ support will diminish. Previous research found that LTBI treatment completion rates are higher for patients treated by providers who prescribed frequently [37], which indicates that growing experience of primary care providers in treating LBTI may eventually result in better treatment completion rates. That said, consolidating relationships between tertiary and primary care providers would be an important ongoing consideration in setting up LTBI management in primary care.

At the organisational level, the availability of dedicated resources to deliver and support patient education and follow-up was inconsistent across the participating sites. The statistically significant difference noted in the completion rate between the two sites may be largely attributed to this discrepancy in resource availability. This suggests that consistent and ongoing patient education and follow-up may have facilitated patient compliance and ultimately treatment success. This is consistent with the findings of a previous study in which nurse led case management combined with education and tracking was found to improve adherence to LTBI treatment in the homeless population [38]. Conceivably, scoping of resources to enable patient education and follow-up might be one means of identifying primary care settings that are most suited to managing LTBI.

Despite the challenges, this study suggests that successful management of LTBI in primary care is achievable. Strategic partnerships, comprising of support from a local tertiary service provider and SEMPHN, were instrumental to the implementation of the LTBI Primary Care Model within the universal primary care setting. Enablers common to both pilot settings, such as familiarity with cultural and clinical aspects relevant to the refugee patient group, multilingual proficiency and interpreter use, free access to medication, and primary care provider training were found to be critical for the sustainable treatment of LTBI patients and should be considered in setting up LTBI management in primary care.

## Limitations

The small size of the study population did not allow detailed investigation into factors associated with higher treatment completion rates in the refugee-focused clinic compared with the universal primary care clinic. While the qualitative data suggest that limited organisational resources in the universal primary care setting may have contributed to challenges in treatment completion, further research is needed to confirm this. In addition, this study explored patient-level barriers from the perspective of health providers, rather than patients themselves. Future qualitative research could explore identified issues, such as motivation and health literacy, from the patients’ perspectives. This could be valuable for ensuring and improving the ‘patient-centredness’ of the LTBI Primary Care Model. The research team consisted of MHRHW employees. To ensure that their affiliation does not impact the evaluation objectivity, the researchers critically examined their own role, potential bias and influence during formulation of the research questions, data collection and interpretation.

### Sustainability

Despite the challenges encountered in treating LTBI in primary care settings, particularly universal primary care, patient management has been continued at both pilot sites. Relationships established through the pilot have been sustained, with MHRHW infectious diseases physicians providing ongoing support to the universal primary care clinic. Additional clinical support has been provided by the Victorian TB Program. Medication is available through the Victorian TB Program at no cost, however, ongoing challenges in addressing private dispensing costs will need to be negotiated. This has relevance to some cohorts of people of refugee background due to restrictions on work rights and limited financial support. For this pilot, ongoing costs for the GP, ID specialist and RHN consultations have been covered through the existing budgets of the participating organizations. However, upscaling the pilot will require resources to fund dedicated clinical staff for patient education and follow-up.

## Conclusion

Despite LTBI having been traditionally managed in the context of tertiary outpatient settings, this project confirms that screening and management can be achieved within primary care, considerate of barriers and enablers at patient, provider, organisational and clinical levels. Patient related factors and limitations of the LTBI medication (isoniazid) confirm that LTBI infection can be difficult to manage regardless of the setting. However, treatment adherence and thereby successful outcomes can be improved by consolidating relationships between tertiary and primary care settings, thus, facilitating primary care provider training and support through secondary infectious diseases consultations. Successfully upscaling the primary care response will require supporting primary care clinics with additional resources, in particular, dedicated clinical staff for patient education, follow-up communication, and monitoring medication adherence and adverse reactions.

## Supplementary Information

**Additional file 1.** Patient Education Resource Pack.

## Data Availability

Clinical data generated from patients and qualitative data generated from service providers are not publicly available due to privacy and ethical concerns. The datasets used and/or analysed during the current study are available from the corresponding author on reasonable request. Materials generated to facilitate LTBI patients’ education and follow-up are available in the (Additional file 1. Patient Education and Resource Pack).

## Abbreviations

CPD: Continuous Professional Development
GP: General Practitioner
LTBI: Latent tuberculosis infection
MHRHW: Monash Health Refugee Health and Wellbeing
PBS: Pharmaceutical Benefits Scheme
RACGP: Royal Australian College of General Practitioners
RHN: Refugee health nurse
SEMPHN: South Eastern Melbourne Primary Health Network
