# Supplementary material for: Improving screening and management of latent tuberculosis infection: development and evaluation of latent tuberculosis infection primary care model
Source: BMC Infect Dis. 2022 Jan 12;22:49. doi: 10.1186/s12879-021-06925-8 (PMC8756639; doi:10.1186/s12879-021-06925-8)
Supplement: Supplementary file 1 — Additional file 1. Patient Education Resource Pack. [file 12879_2021_6925_MOESM1_ESM.pdf]

Improving screening and management of Latent Tuberculosis Infection: development and evaluation of Latent Tuberculosis Infection Primary Care Model

Marina Kunin[1],

Mark Timlin[1],

Chris Lemoh[1,2],

David A. Sheffield[1,2],

Alana Russo[1]

Shegofa Hazara [1]

Jacquie McBride[1]

1. Monash Health Refugee Health and Wellbeing, Monash Health, 122 Thomas Street, Dandenong, Vic. 3175, Australia.
2. Monash Infectious Diseases, Melbourne, VIC, Australia.

Corresponding author: Marina Kunin, PhD, Monash Health Refugee Health and Wellbeing, Monash Health, 122 Thomas Street, Dandenong, Vic. 3175, Australia. Email: marina.kunin@monashhealth.org.au

Appendix 1.1

LATENT TUBERCULOSIS TREATMENT

This information is for patients, families and carers.

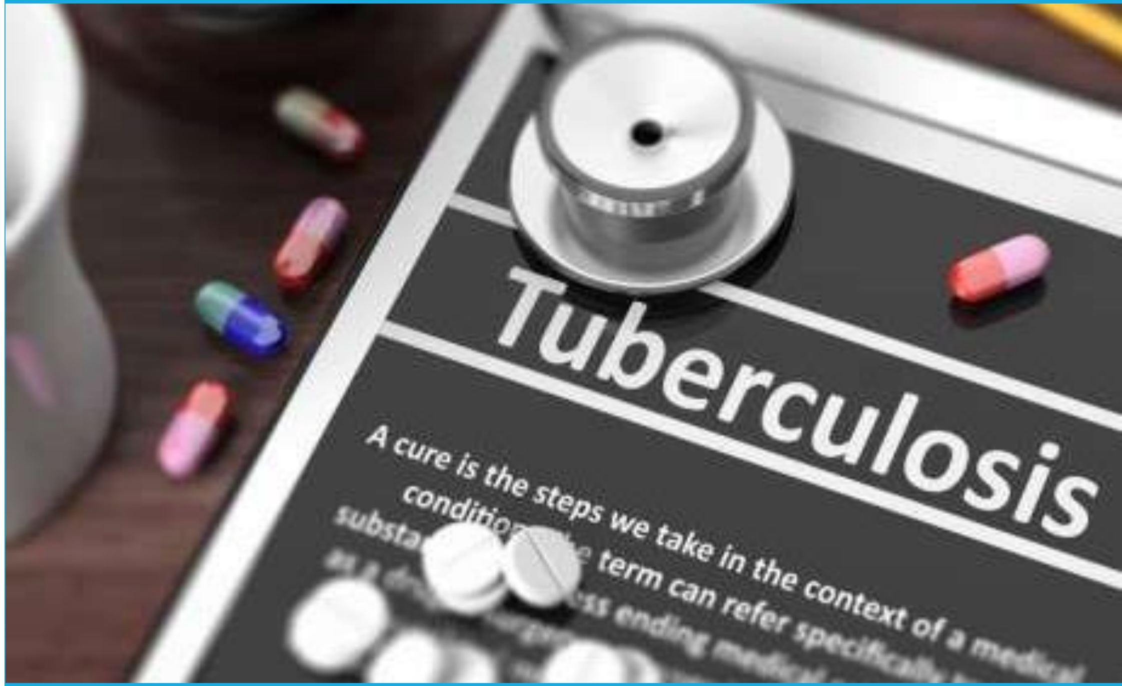

MonashHealth

Patient details

Name:	……………………………………………..
Date of birth:	……………………………………………..
Address:	……………………………………………..
Language:	……………………………………………..
Treatment start date:	……………………………………………..
Treatment completion date:	……………………………………………..

MonashHealth
Refugee Health and Wellbeing

GP contact details

GP name: ……………………………………………..

Clinic: ……………………………………………..

Phone: ……………………………………………..

Address: ……………………………………………..

……………………………………………..

……………………………………………..

Refugee Health contact details

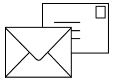

Nurse Name
Refugee Health and Wellbeing
Monash Health

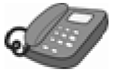

Phone:

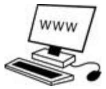

www.monashhealth.org

What your medications look like and when to take them

<table>
<tr>
<td>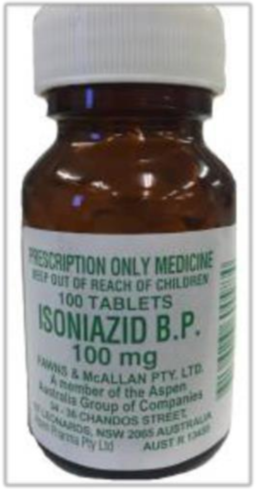
</td>
<td>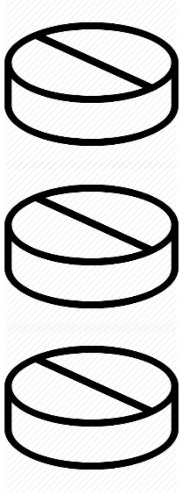</td>
<td>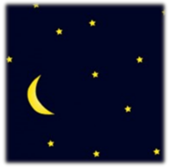
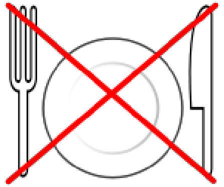
Empty stomach</td>
</tr>
</table>

<table>
<tr>
<td>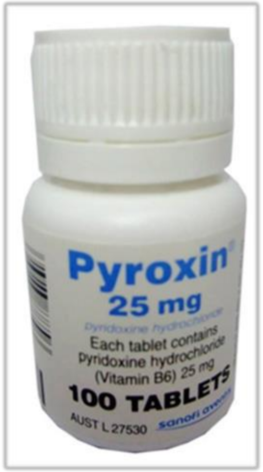
</td>
<td>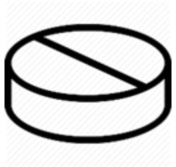</td>
<td>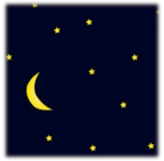
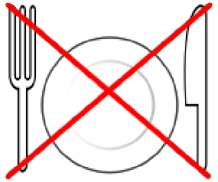
Empty stomach</td>
</tr>
</table>

2017

- Place O on the days medication taken
- Place X when next medication pick-up is due from pharmacy

January						
M	T	W	T	F	S	S
						1
2	3	4	5	6	7	8
9	10	11	12	13	14	15
16	17	18	19	20	21	22
23	24	25	26	27	28	29
30	31					

February						
M	T	W	T	F	S	S
		1	2	3	4	5
6	7	8	9	10	11	12
13	14	15	16	17	18	19
20	21	22	23	24	25	26
27	28					

March						
M	T	W	T	F	S	S
		1	2	3	4	5
6	7	8	9	10	11	12
13	14	15	16	17	18	19
20	21	22	23	24	25	26
27	28	29	30	31		

April						
M	T	W	T	F	S	S
					1	2
3	4	5	6	7	8	9
10	11	12	13	14	15	16
17	18	19	20	21	22	23
24	25	26	27	28	29	30

May						
M	T	W	T	F	S	S
1	2	3	4	5	6	7
8	9	10	11	12	13	14
15	16	17	18	19	20	21
22	23	24	25	26	27	28
29	30	31				

June						
M	T	W	T	F	S	S
			1	2	3	4
5	6	7	8	9	10	11
12	13	14	15	16	17	18
19	20	21	22	23	24	25
26	27	28	29	30		

- **If you have any of the listed symptoms, see your GP within 24 hours**
- **If you are not able to see your GP within 24 hours, go to nearest Emergency Department**

Possible medication side effects

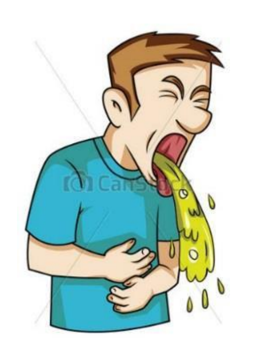

Nausea/vomiting

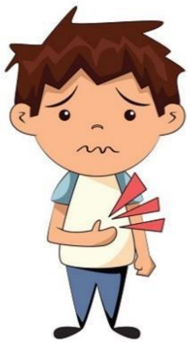

Stomach pain

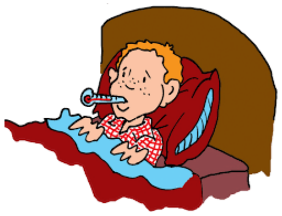

Hot and sweaty

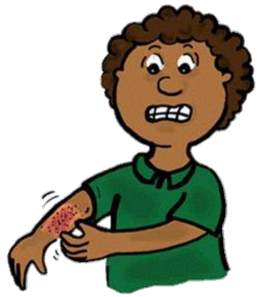

Skin rash

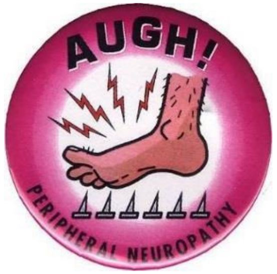

Tingling in fingers and toes

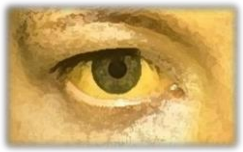

Yellowing of eyes and skin

2017

July

M	T	W	T	F	S	S
					1	2
3	4	5	6	7	8	9
10	11	12	13	14	15	16
17	18	19	20	21	22	23
24	25	26	27	28	29	30
31						

August

M	T	W	T	F	S	S
	1	2	3	4	5	6
7	8	9	10	11	12	13
14	15	16	17	18	19	20
21	22	23	24	25	26	27
28	29	30	31			

September

M	T	W	T	F	S	S
				1	2	3
4	5	6	7	8	9	10
11	12	13	14	15	16	17
18	19	20	21	22	23	24
25	26	27	28	29	30	

October

M	T	W	T	F	S	S
						1
2	3	4	5	6	7	8
9	10	11	12	13	14	15
16	17	18	19	20	21	22
23	24	25	26	27	28	29
30	31					

November

M	T	W	T	F	S	S
		1	2	3	4	5
6	7	8	9	10	11	12
13	14	15	16	17	18	19
20	21	22	23	24	25	26
27	28	29	30			

December

M	T	W	T	F	S	S
				1	2	3
4	5	6	7	8	9	10
11	12	13	14	15	16	17
18	19	20	21	22	23	24
25	26	27	28	29	30	31

2018

- Place O on the days medication taken
- Place X when next medication pick-up is due from pharmacy

January

M	T	W	T	F	S	S
1	2	3	4	5	6	7
8	9	10	11	12	13	14
15	16	17	18	19	20	21
22	23	24	25	26	27	28
29	30	31				

February

M	T	W	T	F	S	S
			1	2	3	4
5	6	7	8	9	10	11
12	13	14	15	16	17	18
19	20	21	22	23	24	25
26	27	28				

March

M	T	W	T	F	S	S
			1	2	3	4
5	6	7	8	9	10	11
12	13	14	15	16	17	18
19	20	21	22	23	24	25
26	27	28	29	30	31	

April

M	T	W	T	F	S	S
						1
2	3	4	5	6	7	8
9	10	11	12	13	14	15
16	17	18	19	20	21	22
23	24	25	26	27	28	29
30						

May

M	T	W	T	F	S	S
	1	2	3	4	5	6
7	8	9	10	11	12	13
14	15	16	17	18	19	20
21	22	23	24	25	26	27
28	29	30	31			

June

M	T	W	T	F	S	S
				1	2	3
4	5	6	7	8	9	10
11	12	13	14	15	16	17
18	19	20	21	22	23	24
25	26	27	28	29	30	

2018

July

M	T	W	T	F	S	S
						1
2	3	4	5	6	7	8
9	10	11	12	13	14	15
16	17	18	19	20	21	22
23	24	25	26	27	28	29
30	31					

August

M	T	W	T	F	S	S
		1	2	3	4	5
6	7	8	9	10	11	12
13	14	15	16	17	18	19
20	21	22	23	24	25	26
27	28	29	30	31		

September

M	T	W	T	F	S	S
					1	2
3	4	5	6	7	8	9
10	11	12	13	14	15	16
17	18	19	20	21	22	23
24	25	26	27	28	29	30

October

M	T	W	T	F	S	S
1	2	3	4	5	6	7
8	9	10	11	12	13	14
15	16	17	18	19	20	21
22	23	24	25	26	27	28
29	30	31				

November

M	T	W	T	F	S	S
			1	2	3	4
5	6	7	8	9	10	11
12	13	14	15	16	17	18
19	20	21	22	23	24	25
26	27	28	29	30		

December

M	T	W	T	F	S	S
					1	2
3	4	5	6	7	8	9
10	11	12	13	14	15	16
17	18	19	20	21	22	23
24	25	26	27	28	29	30
31						

Appendix 1.2

LTBI Patient Education Session Checklist

- What is tuberculosis (TB)
- How TB is transmitted and areas most likely to be affected
- Difference between Tuberculosis disease (Active TB) and risk of developing TB disease (latent TB)
- Changes in the CXR – if have been exposed/ infective in the past
- The risk of reactivation and lifelong risk percentage
- Co-morbidities/risk factors e.g. Hepatitis, HIV
- Treatment and possible side effects
- If treatment is declined – the need for 6 monthly CXR for 2 years post positive test result
- Risk of secondary infection
- LTB and treatment in pregnancy and while breastfeeding
- Signs and symptoms of active TB
- Productive cough
- Haemoptysis (coughing up blood)
- Fever, night sweats
- Unexplained weight loss
- Unexplained loss of appetite
- To attend the nearest emergency department if any signs and symptoms of active TB
- Once on treatment – ongoing nursing and nursing review needed
- To contact clinic if any issues – missed dose, lost script or pathology slips

Appendix 1.3

Name:
Address:

NURSE CHECK-UP SESSION

Latent TB nursing review

Nurse: [ ]

Date: [ ]

1.1.1 Latent TB understanding	1.1.2 Notes
Has pharmacist checked patient understanding?	Yes / No Date:
Understands the difference between active TB and latent TB	Yes / No
Understands rationale of taking medications<br>(To prevent future TB disease)	Yes / No
Knows medication name(s), dose(s) and frequency	Yes / No
Knows their course duration<br>(Planned Duration)	Yes / No Start Date:<br><br>Finish Date:

1.1.3 Adherence		1.1.4 Notes
Method of checking adherence (Check at least 2 checkboxes)	☐ Patient	Number of days missed:
	☐ Check dispensing history	
	☐ Check LTBI calendar or adherence	
	☐ Counted tablets left in bottle	
Reason for non-adherence		

1.1.5 Side-effects		1.1.6 Notes
Allergy? (rash, hives, difficulty breathing etc)	Yes / No If Yes specify:	☐ Refer to doctor
Hepatic side-effects? (yellow skin, yellow eyes, Abdo cramps, Nausea, bruising, bleeding)	Yes / No	☐ Refer to doctor
Signs of Peripheral Neuropathy? (Tingling or numbness of the fingers and toes)	Yes / No	☐ Refer to doctor
Other side-effects?	Yes / No	☐ Refer to doctor

1.1.7 Physical Examination			1.1.8
Physical symptoms? (hemoptysis, night sweats, febrile, weight loss)	Refer to doctor for investigation		
Vital Signs	BP: mmHg	HR: beats/min	
	Temp: ºC	O_2 Sats: %	
	Weight: kg	Height: cm	

1.1.9 Risk Factors		1.1.10
Malnourished?	Yes / No	
Alcohol Consumption?	Yes / No If yes how much:	

1.1.11 Other Concerns from client	1.1.12

1.1.13 Actions/Plan		1.1.14
Latent TB understanding		
Adherence		
Side-effects for investigation		
Physical Assessment		
Other		

1.1.15 Appointments/Follow-up		1.1.16 Notes
Next GP review		
Next ID review		
Next Nursing Review		
Next Bloods review		
Next new prescription		
